# Electronic aggregated data collection on cervical cancer screening in Bangladesh since 2014: what the data tells us?

**DOI:** 10.1186/s12889-023-17545-z

**Published:** 2024-01-23

**Authors:** Ashrafun Nessa, Md Shahadat Hossain, Sheikh Md Nazim Uddin, Md Rafiqul Islam, Muhammad Abdul Hannan Khan, Abul Kalam Azad

**Affiliations:** 1 Bangabandhu Sheikh Mujib Medical University, Dhaka, Bangladesh; 2 Directorate General of Health Services, Dhaka, Bangladesh; 3 Ministry of Health and Family Welfare, Dhaka, Bangladesh

**Keywords:** Cervical cancer, Cervical cancer screening, VIA, DHIS2 in Bangladesh, Electronic data tracking

## Abstract

**Introduction:**

To reduce the high prevalence of cervical cancers among the Bangladeshi women, the Government of Bangladesh established a national cervical cancer screening programme in 2005 for women aged 30 to 60 years. The District Health Information System Version 2 (DHIS2) based electronic aggregated data collection system is used since the year 2013. This study summarises data from the year 2014 to 2022 to assess the effectiveness of the electronic data collection system in understanding the outcome of the screening programme.

**Methods:**

This is a descriptive study based on secondary data extracted in MS Excel from the DHIS2-based electronic repository of the national cervical cancer screening programme of Bangladesh. The respondents were women aged 30–60 years, screened for cervical cancer using VIA (Visual Inspection of cervix with Acetic acid) method in 465 government health facilities. The data were collected on the participants’ residential location, month and year of screening, name and type of health facilities performing VIA, and VIA screening results.

**Results:**

The national screening programme reported a total 3.36 million VIA tests from 465 government hospitals in 8 years (2014 to 2022). The national average VIA-positivity rate was 3.6%, which varied from 1.4 to 9.5% among the districts. This national screening programme witnessed an exponential growth, year after year, with 83.3% increase in VIA test from 2014 to 2022. The primary and the secondary care hospitals were the highest collective contributors of VIA tests (86.2%) and positive cases (77.8%). The VIA-positivity rates in different hospital types varied widely, 7.0% in the medical university hospital, 5.7% in the medical college hospitals, 3.9% in the district/general hospitals, and 3.0% in the upazila health complexes.

**Conclusions:**

A national cervical cancer screening programme using VIA method and a DHIS2-based electronic data collection backbone, is effective, sustainable, and useful to understand the screening coverage, VIA positivity rate and geographic distribution of the participants and case load to initiate policy recommendations and actions. Decentralization of the screening programme and more efforts at the primary and secondary care level is required to increase screening performances.

## Introduction

Cervical cancer (CC) is one of the most prevalent cancers among Bangladeshi women. There were approximately 8,268 new CC cases in the year 2020 with an age-standardised incidence rate of 10.6 and mortality rate of 6.67 per 100,000 population [1]. Due to national commitment of improving the health of the women [2] and taking the advantage of the extensive healthcare delivery network from national to subnational levels, the Ministry of Health and Family Welfare (MOHFW) launched a national cervical cancer screening programme for women aged 30 to 60 years in 2005 using Visual Inspection of Cervix with Acetic Acid (VIA) method [3–5].

An electronic system for collection of monthly aggregated data from the participating national to subnational health facilities were introduced in 2013 [6–9]. The electronic data collection system is built on District Health Information System version 2 (DHIS2), an open-source platform developed by the University of Oslo, used in over 70 Low and Middle-Income Countries (LMICs) [10, 11]. The DHIS2 gained popularity because it has been built to serve the purpose of public health data gathering, validation, analysis, and ready visualization in required level of aggregation and disaggregation [12].

A “National Centre for Cervical and Breast Cancer Screening and Training (NCCBCST)” has been established at Bangabandhu Sheikh Mujib Medical University (BSMMU) for competency-based training of service providers, better coordination, improvement of quality of the screening, and the programme was scaled up to the sub-district level for rapid increase of population coverage [13, 14]. The women, who are found VIA-positive during the screening, are referred for special check-ups and subsequent treatment to the colposcopy clinics available at the designated hospitals across the country. Though the CC screening data are being stored for several years in the DHIS2, adequate evaluation has not been assessed to check it’s usefulness and effectiveness. The objective of this paper is to summarize data for the CC screening from the year 2014 to 2022 to assess the effectiveness of the electronic data collection system in understanding the outcome of the screening programme in Bangladesh.

## Methods

This is a descriptive study based on secondary data extracted from the electronic repository of the national cervical cancer screening programme of Bangladesh, in which the data are collected on monthly basis in aggregated form through a DHIS2-based electronic data collection system. The respondents were women aged 30–60 years, screened for CC using VIA method in 465 government health facilities of the country; comprised of 378 upazila health complexes (UHCs, primary care hospitals), 64 district/general hospitals (DH/GH, secondary care hospitals), 16 medical college hospitals (MCHs), one university hospital (Bangabandhu Sheikh Mujib Medical University Hospital, BSMMU), and 6 other hospitals, viz., the National Institute of Cancer Research and Hospital (NICRH), Institute of Child and Mother Health (ICMH), one 100-bed and three 20–31 bed hospitals. Data were extracted from the year 2014 to 2022 in MS Excel and checked and cleaned for inconsistencies and ambiguity, if any. The data were stored in aggregated form and contained information of the women on geographic location of residence (division, district), month and year of VIA screening, name and type of health facilities where the VIA screening was done and the screening results. Data were analyzed using SPSS (version 23) to see the distribution and disaggregation of the VIA screening tests and their results by geographic location, year, and the health facility types.

## Results

**Fig. 1:**
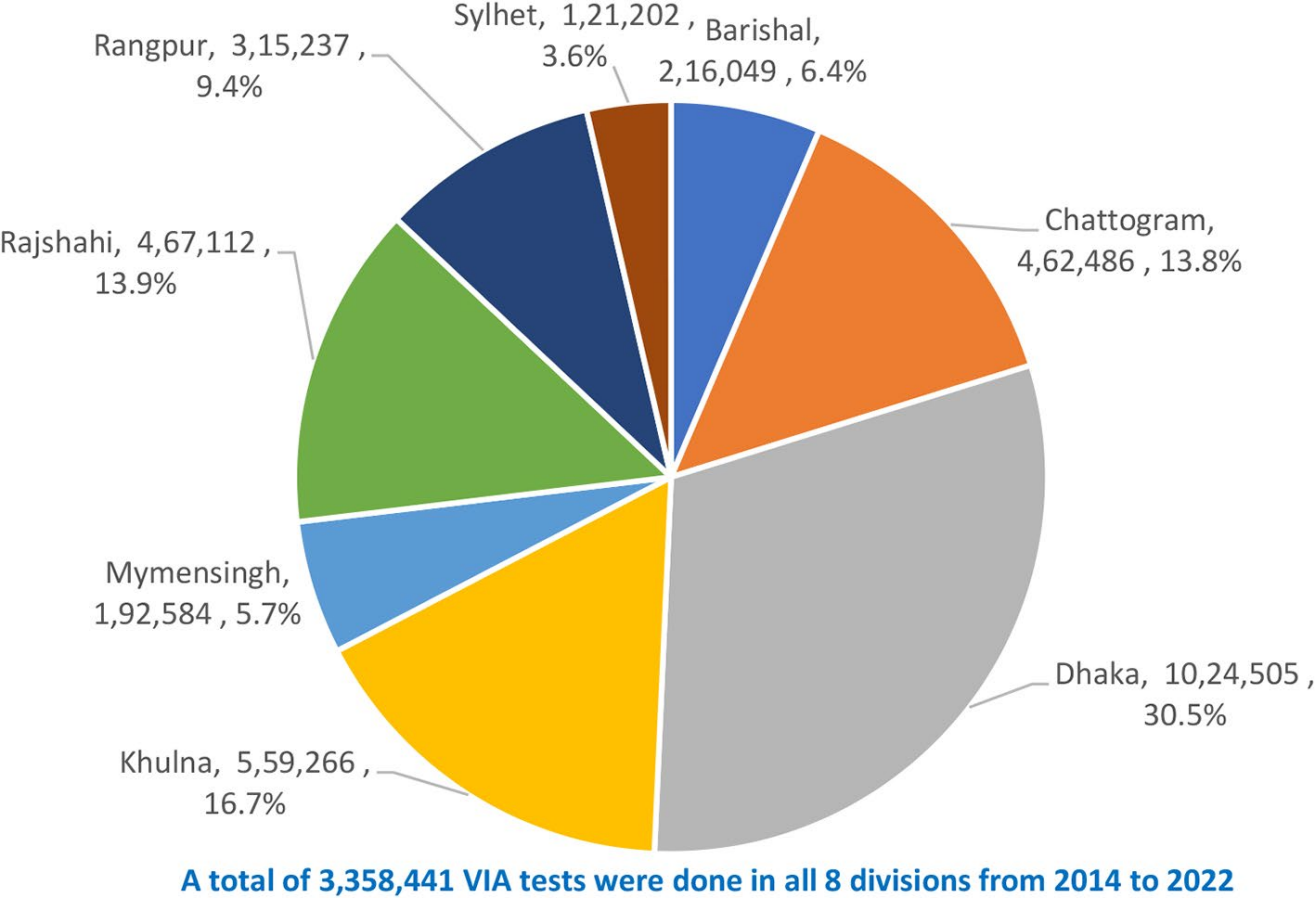
Division-wise distribution of number of VIA tests done from 2014 to 2022 in 8 divisions of Bangladesh

Figure 1 shows the distribution of the cumulative number of VIA tests done in different divisions of Bangladesh between 2014 and 2022. Among the 3,358,441 VIA tests done in the country, maximum number of the tests were done in Dhaka division (1,024,505 tests; 30.5%) followed by Khulna division (559,266 tests; 16.7%). The least number of tests were done in Sylhet division (121,202 tests; 3.6%).

**Fig. 2:**
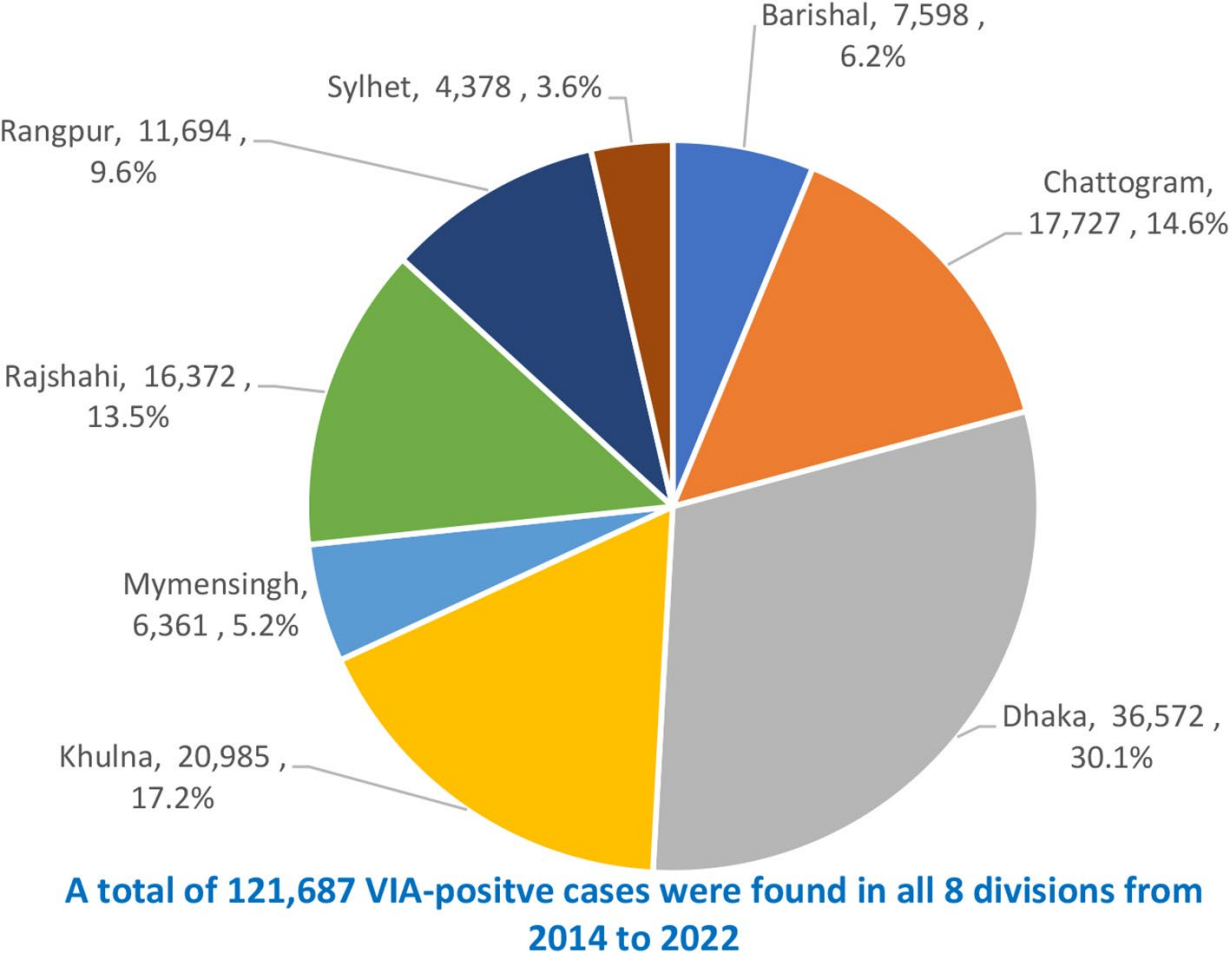
Division-wise distribution of number of VIA-positive cases from 2014 to 2022 in 8 divisions of Bangladesh

Figure 2 shows the distribution of the VIA-positive cases among 8 divisions covering the mentioned period. A total of 121,687 (3.6%) women tested with VIA were found positive. The highest number of VIA-positive cases were in Dhaka division (36,572 cases; 30.1%) followed by Khulna division (20,985 cases; 17.2%) and the least number were in Sylhet division (4,378 cases; 3.6%).

**Fig. 3:**
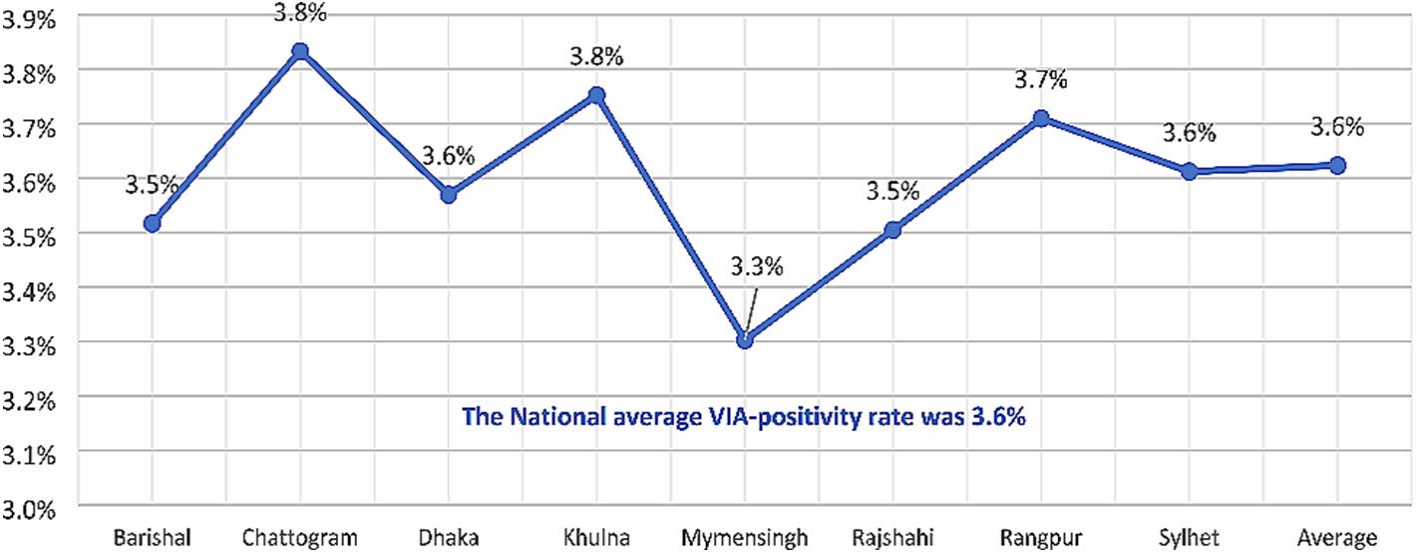
Division-wise VIA-positivity rate from 2014 to 2022 in 8 divisions of Bangladesh

Figure 3 shows the VIA-positivity rate in different divisions. The national average VIA-positivity was 3.6%. The highest positivity rate was found in Chattogram and Khulna divisions (3.8%), followed by in Rangpur (3.7%), Dhaka and Sylhet (3.6%), Barishal and Rajshahi (3.5%), and Mymensingh (3.3%).

**Fig. 4:**
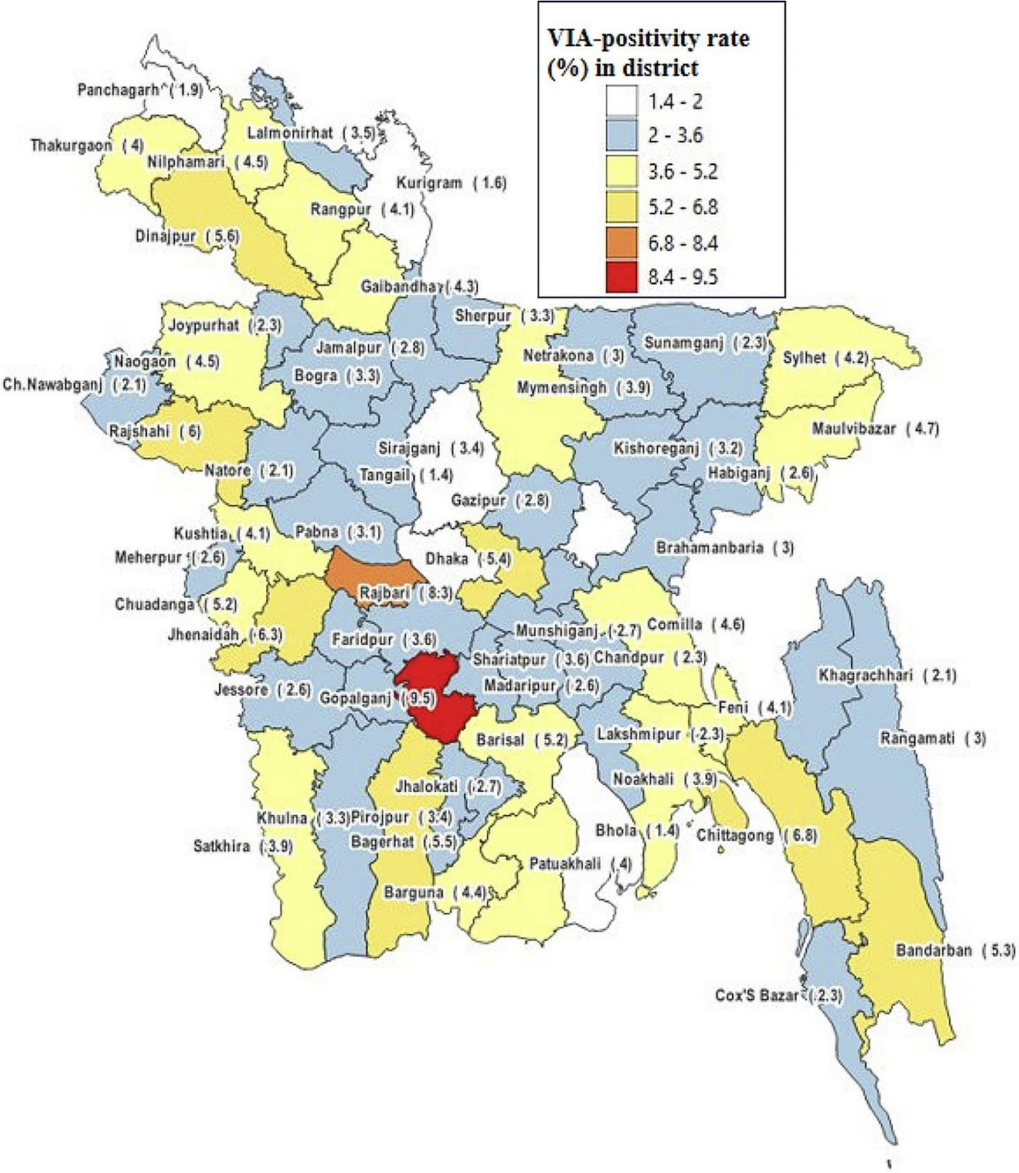
Division-wise distribution of cumulative VIA-positivity rates in map of Bangladesh over 8-years (2014 to 2022)

When the VIA-positivity rate was disaggregated by district (Fig. 4), the rate was varied between 1.4% and 9.5%. In the map of Bangladesh, the districts have been coloured in six groups. The mean ± SD VIA positivity rate in the districts was (3.6 ± 1.6)%. We grouped the districts with respect to their distance from the mean VIA-positivity rate (national average) through subtracting or adding the standard deviation. It revealed that the VIA-positivity rate in Gopalganj district was exceptionally high (9.5%, more than 3SDs above the mean). In Rajbari district, the rate was 8.3% (more than 2SDs above the mean). Six districts, viz., Chittagong, Bandarban, Bagerhat, Jhenaidah, Rajshahi, and Dinajpur had the VIA-positivity more than 1SD above the mean.

**Fig. 5:**
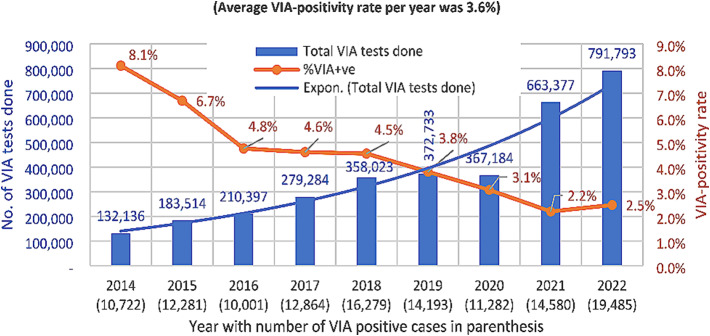
Year-wise distribution of number of VIA tests and VIA-positive cases in 8 divisions of Bangladesh (from 2014 to 2022)

Figure 5 shows the year-wise distribution of number of VIA tests and VIA-positivity rate reported from all eight administrative divisions of Bangladesh. In general, each subsequent year, the number of VIA tests increased than in the previous year in exponential phenomenon. The highest number of tests were done in 2022 (791,793 tests) and the lowest number of tests were done in 2014 (132,136 tests), an 83.3% growth. The average VIA-positivity rate over the years was 3.6%. The VIA-positivity was highest in the year 2014 (8.1%), which declined gradually in the subsequent years. In 2022, the VIA-positivity was 2.5%, and the lowest was in 2021 (2.2%).

**Fig. 6:**
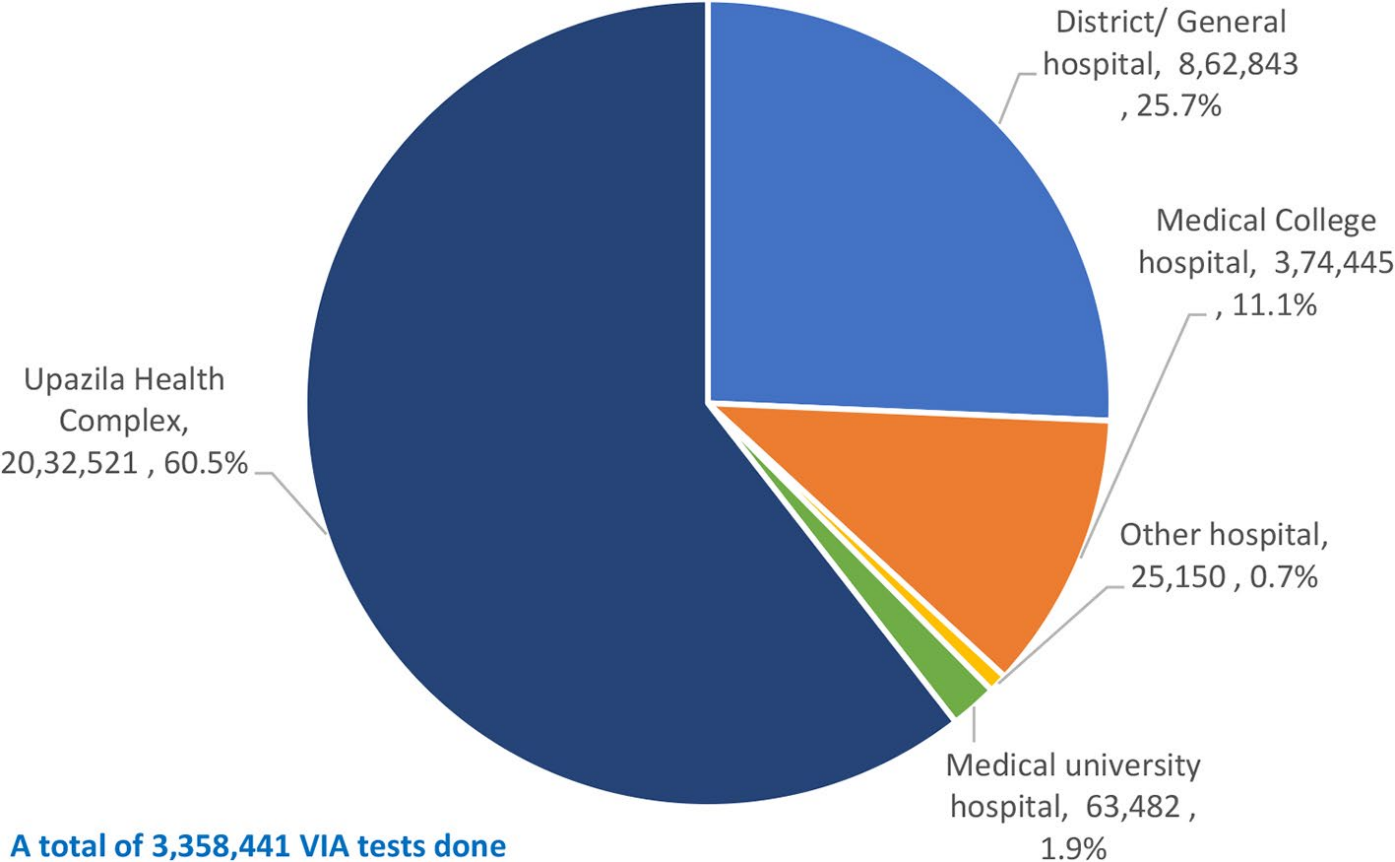
Distribution of number of VIA tests by health facility type (465 hospitals)

Figure 6 shows the distribution of number of VIA tests done from 2014 to 2022 in different type of health facilities. About two-thirds (60.5%; 2,032,521 test) of the tests were done in the UHCs, over a quarter (25.7%; 862,843 tests) were done in the DHs/GHs, 11.1% (374,445 tests) were done in the MCHs, 1.9% (63,482 tests) were done in the university hospital, and 0.7% (25,100 tests) were done in 5 other hospitals.

**Fig. 7:**
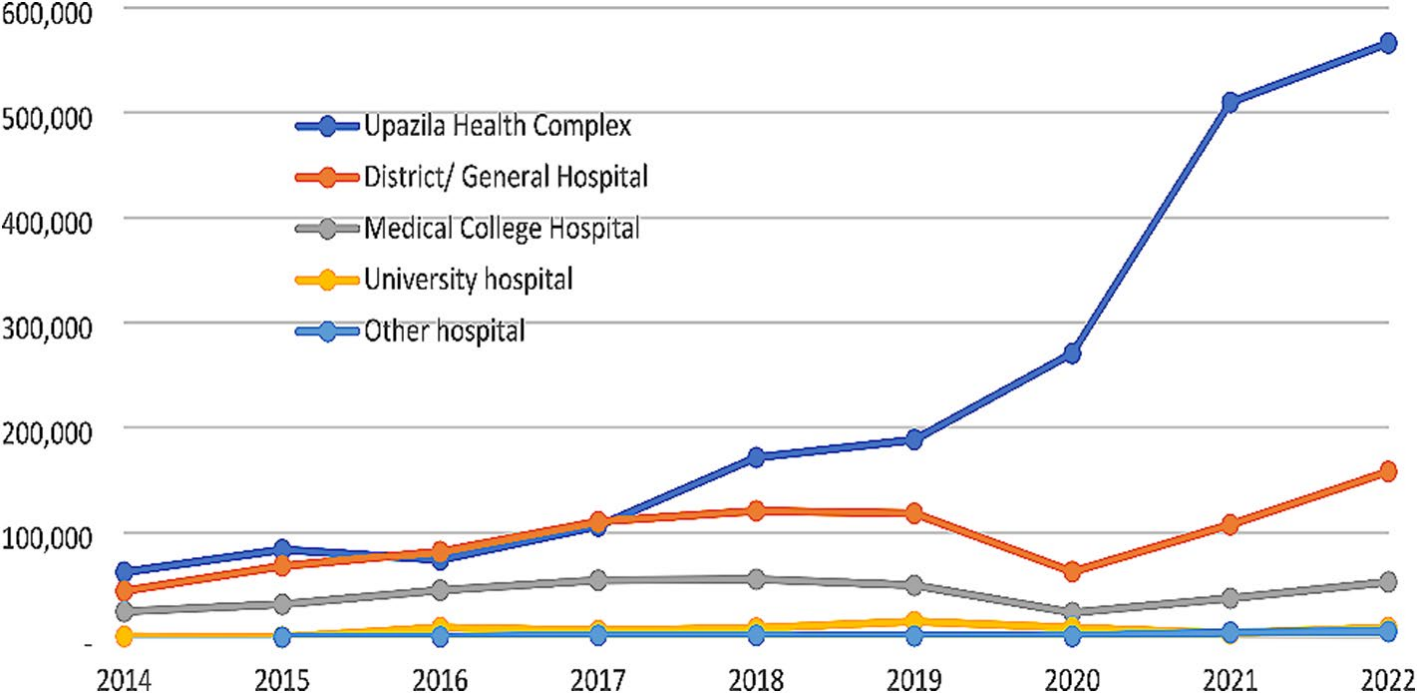
Year-wise distribution of number of VIA tests done by health facility type (465 hospitals)

Figure 7 shows the year-wise trend of VIA tests done in different type of health facilities. The number of VIA tests done consistently increased in each type of health facilities in the subsequent years in comparison to the previous year. However, a drop was seen in 2020 compared to 2019. In the UHCs, although there was no drop, the increase was small.

**Fig. 8:**
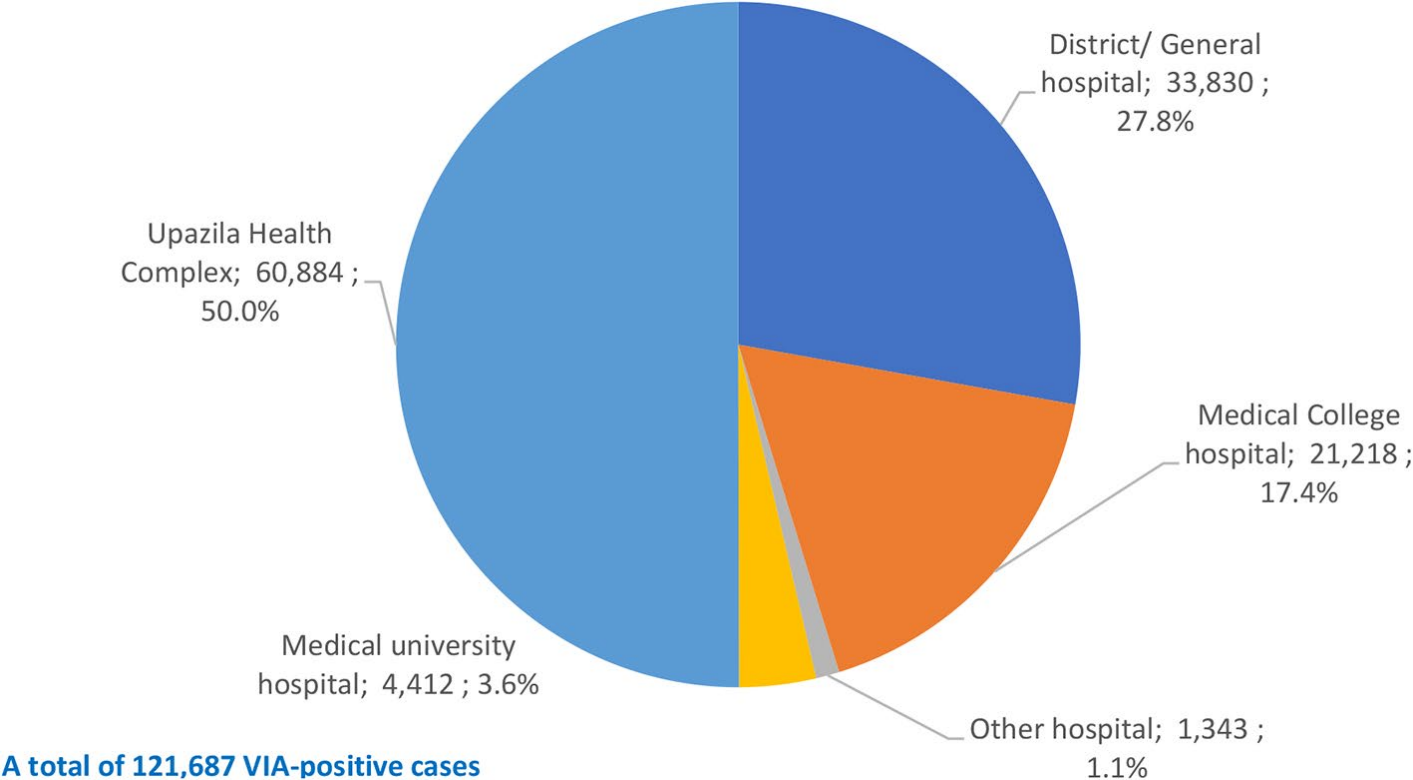
Distribution of number of VIA-positive cases by health facility types (465 hospitals)

Figure 8 shows the distribution of number of VIA-positive cases by different types of health facilities. Of the total of 121,687 VIA-positive cases identified in 8 years, half (60,884 tests; 50.0%) were identified in the UHCs; over one-quarter (33,830 tests; 27.8%) in the DHs/GHs; 17.4% (21,218) in the MCHs, 3.6% (4,412) in the medical university hospital and 1.1% (1,343) tests in the other hospitals.

**Fig. 9:**
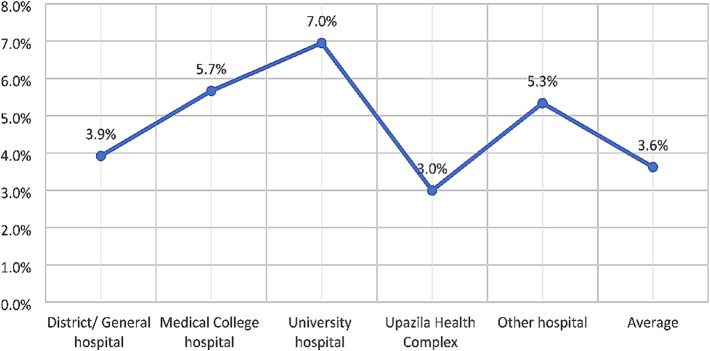
VIA-positivity rate in different types of health facilities (465 hospitals)

The average VIA-positivity rate during the 8-year period in different types of health facilities varied widely (Fig. 9). While the national average rate was 3.6% for all types of hospitals, it was higher in the medical university hospital (7.0%), MCHs (5.7%), DHs/GHs (3.9%), and in the other hospitals (5.3%). At the UHC level the VIA-positivity (3.0%) was lower than the national average.

## Discussions

This study used the secondary source of electronic aggregated data extracted from the repository of the national cervical cancer screening programme of Bangladesh. The programme enrols women aged 30 to 60 years and uses VIA tests for the screening of the cervixes for any suspicion of presence of CC or pre-cancer. In this study, data from 465 government hospitals covering a period from 2014 to 2022 were included.

The consistent upward trend of CC screening across Bangladesh, engaging most of the relevant government health facilities, and gathering of the data through electronic data collection system as evident in Figs. 5 and 7, indicate that the national cervical cancer screening programme and its DHIS2-based data collection system have become sustainable and effective.

It is encouraging that 3.36 million women aged 30 to 60 years were screened for CC during the 8-year period (2014 to 2022) (Fig. 1), although this number represents only 11.42% of the 29.43 million target women (30–60 years) in Bangladesh (excerpted from Bangladesh Population Census 2022 [15]. The screening coverage in Thailand and England has been reported as 53.9% and 78% respectively [16, 17]. Countries of Sub-Saharan Africa have much lower screening coverage such as 3% in Ghana [18], 4.8% in Uganda [19], and 4.8% in Cameroon [20]. A community-based cross-sectional survey in an urban community of South India revealed 7.1% screening coverage [21]. Although the screening coverage in Bangladesh is higher compared to few developing countries as mentioned above, it is much lower compared to Thailand and England. The Bangladesh data call for rapid scaling of the screening programme and establishing the importance of the electronic data collection system, which is enabling monitoring and understanding the situation for policy recommendation and action. During the COVID pandemic, the electronic information system, tremendously helped to identify the screening gaps in different geographic locations as well in health facilities, enabling interventions to bring back the screening coverage to the reasonable pace, otherwise the drop as seen in 2020 (Figs. 5 and 7) could be further down [22, 23].

The cumulative number of VIA tests in 8-years shows Dhaka division to outperform other divisions representing one-third of the total tests (1,024,505 tests; 30.5% of 3.36 million). This is quite reasonable as Dhaka division represents 27% of the total country population [15], Dhaka is the capital city of Bangladesh having multiple national level sophisticated medical facilities that attract patients from all over Bangladesh, and more women from other parts of Bangladesh outside Dhaka division undertake VIA tests in Dhaka. The second highest number of VIA tests in Khulna division is attributed to more health awareness of the people of this division as revealed in national health surveys [24]. Similarly, the underperformance of VIA testing in Sylhet division correlates overall lower health service performance of this division as found in similar national health surveys [24].

The national average VIA-positivity was 3.6%, which varied from 3.3 to 3.8% between divisions (Fig. 3), but between 1.4% and 9.5% if it is disaggregated by districts (Fig. 4). Observations from Kerala and Andhra Pradesh of India showed VIA-positivity rate as 8.4% and 12.7% respectively [25, 26]. In Addis Ababa of Ethiopia and in Sudan, the VIA-positivity rates were 10.3% and 12.7% respectively [27, 28]. Therefore, the overall VIA-positivity in Bangladesh is comparatively lower than several Asian and African countries. In this study, 56 districts, out of 64 showed VIA-positivity rate below 5.2%, 6 districts showed between 5.2% and 6.8%, one district showed 8.3% and only one district showed 9.5% VIA-positivity rate (Fig. 4). This regional variation among the districts in Bangladesh may provide important insight for the policy makers.

The cervical cancer screening programme gained a huge momentum and an exponential growth by 83.3% in the number of VIA tests from the year 2014 to the year 2022 (132,136 vs. 791,793) (Fig. 5). The VIA-positivity rate dropped gradually in these years (8.1% in 2014 and 2.5% in 2022) (Fig. 5). The attributes were multiple. In the initial years, fewer number of women having higher risks of CC or pre-cancer entered the screening programme. During this time, most of the VIA tests were done in the district hospitals and above. In the subsequent years, the programme was gradually rolled out in the primary care level. The UHCs became the major contributors through entry of more and more women irrespective of risk factors because of ease and availability of services close to their homes. The health personnel, over the years, also received multiple hands-on trainings and gained experience and skills.

Of the total 3.6 million VIA tests, 60.5% were done in the UHCs (Fig. 6), contributed to half (60,884; 50.0%) of the VIA-positive cases (Fig. 8). These hospitals (378 UHCs) are primary care hospitals located at sub-districts and close to homes of the women. So, high volume of their collective contribution is obvious. Likewise, DHs/GHs (secondary care hospitals) are the next tier hospitals located at the districts and made a collective contribution of 25.7% of VIA tests with over one-quarter (33,830; 27.8%) of the VIA-positive cases. If these two groups of hospitals are considered together, they contributed to 86.2% of the VIA tests and 77.8% of the VIA-positive cases. These evidences prove that decentralization of the CC screening programme down to the primary and secondary care levels are more effective and more efforts to these centres are needed to enhance coverage.

From Fig. 9, it is found that although the average VIA-positivity rate was 3.6%, it was high in the medical university hospital (7.0%) and MCHs (5.7%). These two types of hospitals are referral hospitals and receive more women with signs and symptoms of diseases attributing to such findings. The 3.0% and 3.9% VIA-positivity rates for UHCs and DHs/GHs are rational as they receive women irrespective of risk factors and perform VIA tests in bulk numbers. The 5.3% VIA-positivity rate by the other hospitals was largely contributed by the high positivity rate found in the National Cancer Institute Hospital, as this is the specialised hospital dealing with cancer patients. The limited set of variables used in the aggregated data system did not allow further data analysis and was an important limitation of this study. Moreover, the aggregated dataset didn’t include the pathological biopsy results of the VIA-positive patients who were referred for colposcopy. Therefore, the authors could not evaluate the accuracy of the VIA test in this program and this was another limitation of this study.

This screening program is a significant part of the primary health care services, strongly linked with Health Management Information System (HMIS) enabling better implementation and assessment through accessibility of timely data and evidence generation. Other studies also focused on the vital role of HMIS in strengthening health systems, assisting patient and programme management, and improving data surveillance [29, 30] with continuous efforts to improve data quality, coordination between programme managers, service providers, data officers, etc. The supervision by local and national level health managers ensures monitoring of data quality using DHIS2 contributes to improve coordination and quality of screening and helps to sustain the screening programme. The WHO has set a CC screening target to achieve at least 70% of screening using a high-performance test (HPV test) at 35 years and again at the age of 45 years within the year 2030 [31]. Bangladesh is continuing VIA test as per the cervical cancer prevention strategy of the country [32]. However, an HPV screening pilot is expected to be launched soon to assess feasibility of rolling it out in the entire country [33] and the existing data in DHIS2 will help in the planning and implementation.

## Conclusions

The VIA-based national cervical cancer screening programme in Bangladesh with a DHIS2-based electronic data collection system, through its several years’ of experience has proven its sustainability and effectiveness. The system is useful to understand the screening coverage, VIA-positivity rate and geographic distribution of the case load to initiate policy recommendations and actions. Decentralization of the screening programme and more efforts at the primary and secondary care level are required to improve screening performances.

## Data Availability

Anonymous summary data are available 24/7 in the programme’s live public dashboard at https://nccbcst.bsmmu.ac.bd/dashboard.

## Abbreviations

CC: Cervical cancer
VIA: Visual Inspection of Cervix with Acetic Acid
DHIS2: District Health Information System version 2
MIS: Management Information System
DGHS: Directorate General of Health Services
MOHFW: Ministry of Health and Family Welfare
UHCs: Upazila Health Complexes.
DHs/GJHs: District/ General Hospitals.
BSMMU: Bangabandhu Sheikh Mujib Medical University.
MCHs: Medical College Hospitals.
NICRH: National Institute of Cancer Research and Hospital.
LMIC: Low and Middle-Income Countries.
HMIS: Health Management Information System.
